# Development of Frequency Based Taste Receptors Using Bioinspired Glucose Nanobiosensor

**DOI:** 10.1038/s41598-017-01855-5

**Published:** 2017-05-09

**Authors:** Amin TermehYousefi, Katsumi Tateno, Samira Bagheri, Hirofumi Tanaka

**Affiliations:** 1 Department of Human Intelligence Systems, Graduate School of Life Science and Systems, Engineering, Kyushu Institute of Technology (Kyutech), 2-4 Hibikino, Wakamatsu, Kitakyushu, 808-0196 Japan; 2 Nanotechnology & Catalysis Research Centre (NANOCAT), IPS Building, University of Malaya, 50603 Kuala Lumpur, Malaysia

## Abstract

A method to fabricate a bioinspired nanobiosensor using electronic-based artificial taste receptors for glucose diagnosis is presented. Fabricated bioinspired glucose nanobiosensor designated based on an artificial taste bud including an amperometric glucose biosensor and taste bud-inspired circuits. In fact, the design of the taste bud-inspired circuits was inspired by the signal-processing mechanism of taste nerves which involves two layers. The first, known as a type II cell, detects the glucose by glucose oxidase and transduces the current signal obtained for the pulse pattern is conducted to the second layer, called type III cell, to induce synchronisation of the neural spiking activity. The oscillation results of fabricated bioinspired glucose nanobiosensor confirmed an increase in the frequency of the output pulse as a function of the glucose concentration. At high glucose concentrations, the bioinspired glucose nanobiosensor showed a pulse train of alternating short and long interpulse intervals. A computational analysis performed to validate the hypothesis, which was successfully reproduced the alternating behaviour of bioinspired glucose our nanobiosensor by increasing the output frequency and alternation of pulse intervals according to the reduction in the resistivity of the biosensor.

## Introduction

Developing taste bud-inspired circuits to designate artificial taste receptors to mimic human abilities has recently become a challenging topic in artificial neural studies focusing on the combination of nanomaterials and electronic science^1, 2^. Previous reports have indicated considerable interest in the integration of human-like nanodevices^3^, especially for those intended for taste sensing capabilities for chemical applications^4^. In this regard, human taste receptors as a whole would be far too complex. Therefore, at the most basic levels, a simpler selective taste receptor without a complicated neural network, such as that of mouse taste buds, is assumed to be a neuron cell that transmits the information signals over a central nervous system and nerve complex^5^. Taste buds endure extreme changes in temperature, pH, and osmolality. Generally, mouse taste bud cells are classified into four cell types: type I, type II, type III, and type IV cells^6, 7^. Type II and type III cells, respectively, contribute primarily to taste reception. In fact, a type II cell includes taste receptors for sweetness, bitterness, and umami, which the given taste bud cells may detect under a noisy environment^8^. Even under such a non-ideal condition, the taste buds perform robust processing of taste information. Such robust information processing with non-uniform sensory units potentially provides useful advancements in bioinspired taste receptors attached to artificial neural networks for human abilities^9^. One of the primary studies in this field of work uses taste bud-inspired circuits for mouse taste bud cells, which is the ultimate example of combination of taste receptors and artificial neurons. Generally, the concentration of chemical components has a direct transient effect on the output frequency of taste nerves^10, 11^, which can be recorded as a signal and transduced via an artificial neural network.

To date, electronic-based artificial taste receptors for the electronic tongue developments are only based on human operator experience. Although the taste bud-inspired circuits utilised in taste receptor design are not exempted, careful rational design can be a foundational approach for the future electronic tongue. Previously, we reported the successful fabrication of an electrochemical enzymatic glucose biosensor using a carbon nanotube composite. Furthermore, a comparative review on enzymatic glucose biosensors using electrochemical methods, especially those fabricated by functionalisation of the carbon nanotube (CNTs)^12^ surface with glucose oxidase (GOx)^13^, was published. In this study, we developed a method to fabricate a bioinspired nanobiosensor for glucose diagnoses following the mouse taste bud network^14^. The proposed bioinspired glucose nanobiosensor consists of two electrical circuits attached to an amperometric glucose biosensor^15^. In the first step, an enzymatic electrochemical glucose biosensor was fabricated based on a CNTs-nanocomposite using our previous reported method. To confirm the biocatalytic activity, the characteristics of the glucose biosensor at optimal conditions were investigated by chronoamperometric measurement. This measurement displayed a representative current–time response for each successive addition of glucose. Then, a continuous pulse was generated by a combination of the fabricated glucose biosensor and taste bud-inspired circuits. The results confirmed that, by adding glucose to bioinspired glucose nanobiosensor, the output pulse frequency moved to a new stable value. However, the high glucose concentration caused an alternate-pulse variation in interpulse intervals. This finding was theoretically reconfirmed by a computational model with the given resistance values as a function of the amount of glucose added to the electrochemical cell. In fact, a simulation was performed to investigate the range of required resistivity of the nanobiosensor to obtain alternations of short and long interpulse intervals. The behaviour displayed by the mathematical model corresponded with the experimental observations. This confirmed that normal periodic pulses were achieved when the resistance of the glucose biosensor was higher than the required resistance for the alternate-pulse variation in the pulse frequency. Normal periodic pulses were achieved in the optimal resistance range, whereas an alternating pulse frequency arises as a consequence of the dynamic instability of the glucose biosensor.

## Fabrication of the glucose biosensor

**Figure 1:**
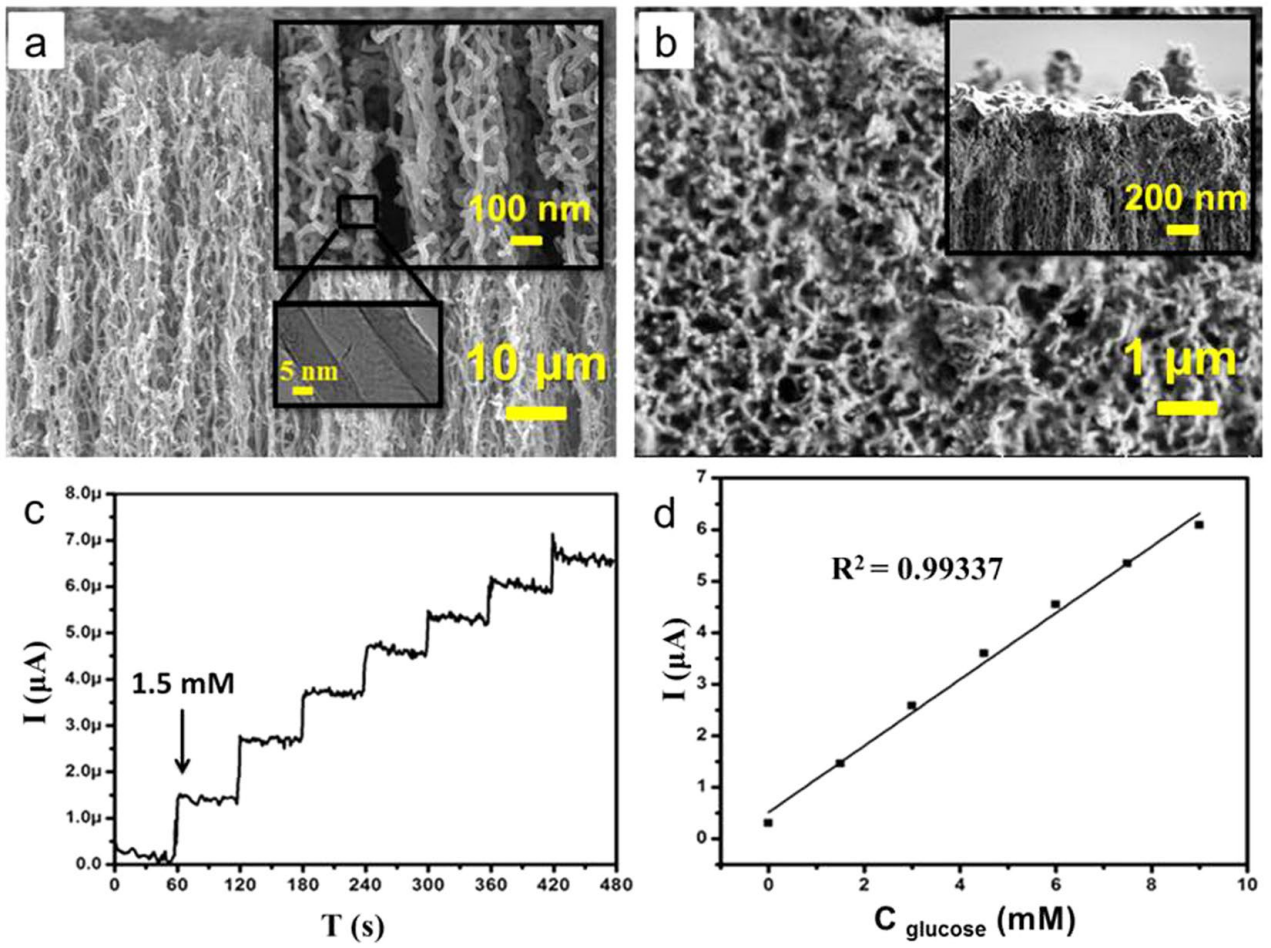
Structural property and chronoamperometric analysis of the enzymatic glucose biosensor. (**a**) FESEM image of synthesised MWCNTs, and (**b**) top view of the composited vertically aligned MWCNTs with Gl and GOx before sonication (inset is the cross section of the composited vertically aligned MWCNTs with Gl and GOx befor sonication). (**c**) Chronoamperometric response of GOx/MWCNTs/Gl/GCE on the successive addition of 1.5 mM glucose in each successive addition at applied potential of +0.3 V. (**d**) Calibration curve of the electrocatalytic current on different concentrations of glucose.

As reported previously^16^, a reagent-free glucose biosensor was prepared based on a vertically aligned multi-walled carbon nanotube (MWNT) composite via an electrochemical method^17^. The defect level of the synthesised MWNTs was in turn successfully optimised by using a chemical vapour deposition (CVD) method^18^. Figure 1(a) indicates the field emission electron microscopy (FESEM) images corresponding to the morphological improvement of the high aspect ratio and uniform MWCNTs. The as synthesised MWCNTs were purified by heating under Ar flow at 600 °C for 100 min and soaked in 6 M of hydrochloric acid (HCl) solution for 1 day. This process was followed by centrifuging^19, 20^. The precipitate was rinsed with deionised water and dried under air. GOx was immobilised on carbon nanotube/gelatin (CNTs/Gl) composite using the entrapment technique. Figure 1(b) demonstrates the FESEM images of the composited vertically aligned MWCNTs via Gl and GOx before sonication.

Approximately 3 µl of MWCNTs/gelatin/GOx was drop casted on GCE and well dried at 4 °C for 12 h. The characteristics of the GOx/MWCNTs/Gl glassy carbon electrode (GCE) at optimal conditions were investigated by chronoamperometric measurement^17^. Figure 1(c) displays a representative current–time response for the successive addition of 1.5 mM of glucose in each successive addition. The calibration curve in Fig. 1(d) with a dynamic linear range spans the glucose concentration range from 1 to 8.9 mM, and it deviates from linearity at higher concentrations, thereby representing a typical Michaelis–Menten kinetics characteristic^21^. The effects of selected possible interference species, such as ascorbic acid, cysteine, uric acid, lactose, and sucrose on the detection of glucose were subsequently investigated. The calculated sensitivity^22^ of the fabricated biosensors was 0.644 µA mM^−1^ by the modified glassy carbon electrode. This value is higher than the sensitivity reported by Cui^23^, Chen^24^, and Ying^22^. The improved sensitivity might have two main reasons: first, the grown MWCNTs were statistically optimised to decrease the number of defects, thereby enhancing the electrical properties of the crystalline MWCNTs^18^. However, MWCNTs with a clean surface present a more effective platform for GOx immobilisation. Second, the amount of immobilized GOx on the surface of the MWCNTs is increased due to the functionalisation process of MWCNTs. Thus, the number of electrons increases due to the presence of a larger amount of GOx for involvement in the glucose detection process. Furthermore, the process by which the enzyme is immobilised on the high-aspect-ratio vertically aligned MWCNTs also has a direct effect on the sensor properties resulting in the micro-environment changing the enzyme and preventing its intrinsic properties, which could improve its affinity to glucose.

## Integration of the taste bud-inspired taste bud circuit: bioinspired glucose nanobiosensor

**Figure 2:**
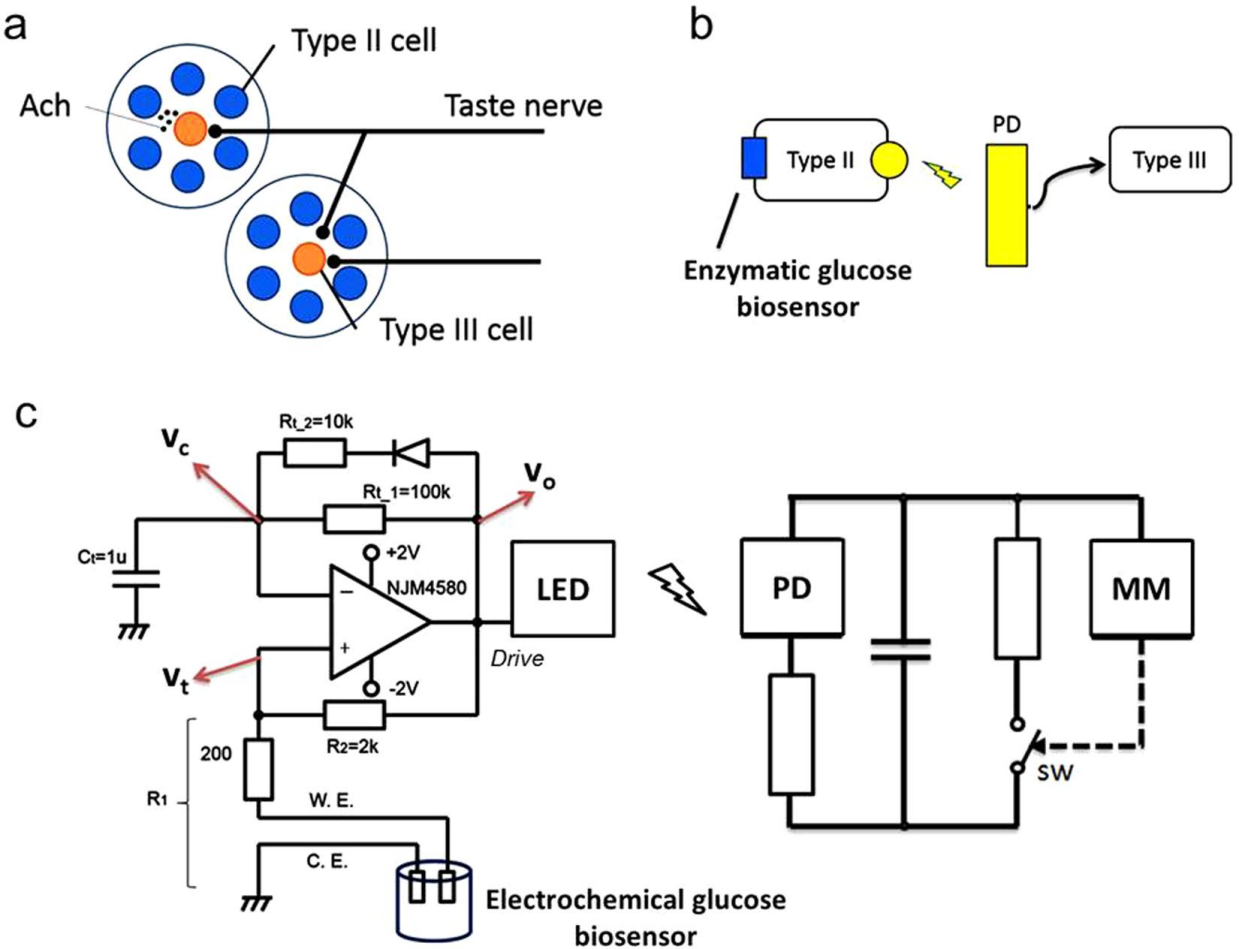
Illustration of electrical circuit attached to glucose biosensor. (**a**) Schematic diagram of mouse taste bud network: type II cells detect chemical components and release signalling molecules, such as Acetylcholine (Ach), into the extracellular space in the taste bud (paracrine signalling). Ach travels to nearby type III cells, which are responsible for transducing the obtained signals to the brain. (**b**) Schematic design of taste bud-inspired circuits: type II cell attached to an amperometric glucose biosensor to obtain signals from the biosensor and transmit signals to the type III cell using light emission. The type II cell circuit produces a signal to induce changes in nearby type III circuit via light emission, as for the paracrine signalling. (**c**) Design of the frequency-based pulse generator circuits includes an infrared LED in the first layer (type II) and an infrared-based photodiode in the second layer (type III) for signal transmission.

According to the glucose detection method, GOx/MWCNTs/Gl/GCE is capable of electrocatalysing the glucose oxidation by using potassium ferricyanide as a mediator in nitrogen-saturated solutions. Therefore, a change in the resistance could be observed with each successive drop of glucose into the electrochemical cell. A previous report on the mouse taste bud network model indicated that the intensity of the chemical stimulus is encoded as the degree of synchronisation of neural pulses^15^. Thus, the bioinspired glucose nanobiosensor was additionally designated to extract and record the change in the resistance in the electrochemical cell and to convert it to generate frequency-based pulses. These pulses were the sum of the transmitted signals between the type II and III cells of the bioinspired glucose nanobiosensor, as shown in Fig. 2(a). Both taste bud-inspired circuits were inspired by the type II and type III cells that exist in the neural network in mouse taste buds. In fact, according to the taste bud network of the mouse, type III cells have synaptic contacts with taste nerve terminals to transmit the signals to the brain, whereas type II cells do not. Hence, in the designated circuits, type II cells were combined with the enzymatic biosensor and the obtained signals transmitted to the type III cells were considered as the output cells.

The bioinspired glucose nanobiosensor was designed to enable the taste bud network to continuously transmit signals to the pattern recognition system. In fact, the type II cell generated pulses in response to the taste stimulation in the first layer. Moreover, the type III cell received the output pulses generated by the type II cell. The sum of neural pulses emitted by independent sources showed an exponential-like distribution of interpulse intervals in the first layer^13, 25^. In other words, the concentration of glucose is detected by the electrochemical glucose biosensor attached to the type II cells, which are responsible to record the alteration in the biosensor resistance and then transduce to the type III cells.

Because of non-physical contact between the respective type II and III cells in the mouse taste bud network, the circuit of the taste bud network was equipped with an infrared LED in the first layer (type II) and infrared-based photodiode in the second layer (type III) to respectively procreate signal identities and transmit the signals produced by the first layer. As shown in Fig. 2(b), the type II cell was responsible for generating repetitive pulses in the first layer. Thus, the output frequency of the type II cell had to increase with an increase in the concentration of glucose detected in the first layer. This finding was validated by theoretical, experimental, and simulation analyses.

## Theoretical analysis of the frequency based taste bud-inspired circuit

**Figure 3:**
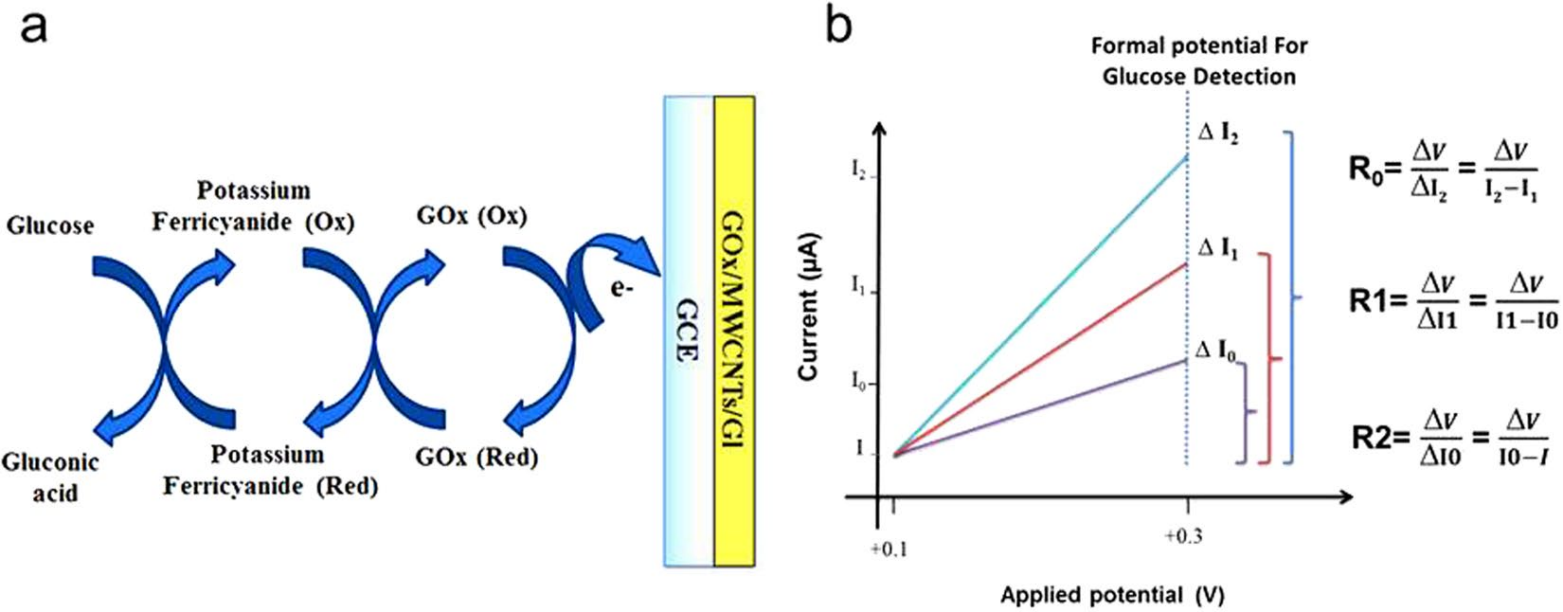
Variation of resistance in the electrochemical cell during glucose detection (**a**) Sequence of events that occur in mediator-based glucose biosensors in the electrochemical cell. (**b**) Output voltage of the pulse generator after the first, second, and third glucose drop into the electrochemical cell.

Figure 3(a) illustrates the mechanism of glucose detection in the electrochemical cell. In the mediator-based glucose biosensor, the mediator electrons shuttle between the active side of the GOx and the electrode surface according to the following scheme:

1 $${\rm{Glucose}}+{\rm{GOx}}({\rm{ox}})\to {\rm{gluconicacid}}+{\rm{GOx}}({\rm{red}})$$

2 $${\rm{GOx}}({\rm{red}})+2{\rm{M}}({\rm{ox}})\to {\rm{GOx}}({\rm{ox}})+2{\rm{M}}({\rm{red}})+2{{\rm{H}}}^{+}$$

3 $$2{\rm{M}}({\rm{red}})\to 2{\rm{M}}({\rm{ox}})+2{{\rm{e}}}^{-}$$

where M(ox) and M(red) are the oxidised and reduced forms of the mediator, respectively.

Electrons that are released from the bioactivity of the enzyme can be received by the working electrode in the electrochemical cell, thereby increasing the current^26^. The alteration of the current created a new resistance value in the electrochemical cell, which could be recorded as frequency in the type II circuit design. Furthermore, the recorded frequency could be transmitted to the type III circuit.

Figure 2(c) shows a pulse generator as a type II cell. The operational amplifier of the type II cell network is NJM4580D (JRC), whereas the output frequency of the pulse generator is determined by resistors (*R*_1_, *R*_2_, *R*_t1_, and *R*_t2_) and a capacitor (*C*_t_). In fact, *R*_1_ provides the resistance between the working electrode and the counter electrode with a fixed small series resistance. Furthermore, the duty ratio of the output pulse was adjusted by a diode and two resistors (*R*_t1_) and (*R*_t2_). The output voltage of the pulse generator circuit was designed for ~1.5 V, which is a relatively high voltage for the electrochemical detection of glucose on account of the presence of the potassium ferricyanide. The resistance of *R*_2_ was roughly adjusted to provide the same resistance as *R*_1_, which reduced the applied voltage to the electrochemical cell to the formal potential of glucose detection. In other words, according to the amperometric characterisations, due to the presence of potassium ferricyanide as a mediator, the detection voltage for glucose is approximately 0.3 V. However, the minimum required voltage for the operational amplifier is higher than the formal potential of the electrochemical cell for glucose detection. Thus, *R*_2_ was included in the first layer of the taste bud-inspired circuit, where it was designated to decrease the input voltage to an appropriate voltage in the electrochemical cell for glucose detection.

Based on the glucose detection mechanism and the continuous variation of resistance in the electrochemical cell^27^, *R*_2_ was also designated in the circuit to record the fraction of resistance in the electrochemical cell. Thus, the addition of glucose to the electrochemical cell changes the resistance of *R*_2_ and the operational amplifier is expected to detect the new voltage of the inverting input (*V*_*t*_). The modified GCE was designed for the continuous detection of glucose because the taste sensory organ identifies sweetness by analysing the unique patterns of responses as a continuous transmission. Thus, the multi-step glucose dropping into the electrochemical cell caused the experiment to generate a different output resistivity according to the glucose concentration, as shown in Fig. 3(b).

The proposed electrical circuit was designed to generate a pulse as a function of the resistance of the electrochemical cell. The output voltage (*V*_*o*_), is altered between a fixed positive value (ON state) and a fixed negative value (OFF state). The voltage difference between the non-inverting and inverting inputs of the operational amplifier (OP amp) determined *V*_*o*_. The inverting input (*V*_*c*_) is the voltage between both ends of capacitor *C*_*t*_. When *V*_*c*_ < *V*_*t*_, *V*_*o*_ is in the ON state; otherwise, it is in the OFF state. In addition, *C*_*t*_ is charged through *R*_*t*2_ when the output voltage was in the ON state, while it is discharged through *R*_*t*1_ when the output voltage is in the OFF state. The charge and discharge cycle determined the pulse frequency. If *R*_1_ is fixed, the output frequency, *f*_pulse_, of the pulse generator is determined by the following equation:

4 $${f}_{{\rm{pulse}}}=\frac{1}{2{R}_{t}{C}_{t}\,\mathrm{ln}(\frac{2{R}_{1}}{{R}_{2}}+1)}$$

where R_1_ is the resistance between the working electrode with MWNTs and the counter electrode, and R_2_ is the fixed resistance. The duty ratio of a pulse was determined by a ratio between the resistors (*R*_*t*1_ and *R*_*t*2_) in the feedback path.

## Electrical circuit of a taste-bud-inspired circuit: single membrane by one GCE

**Figure 4:**
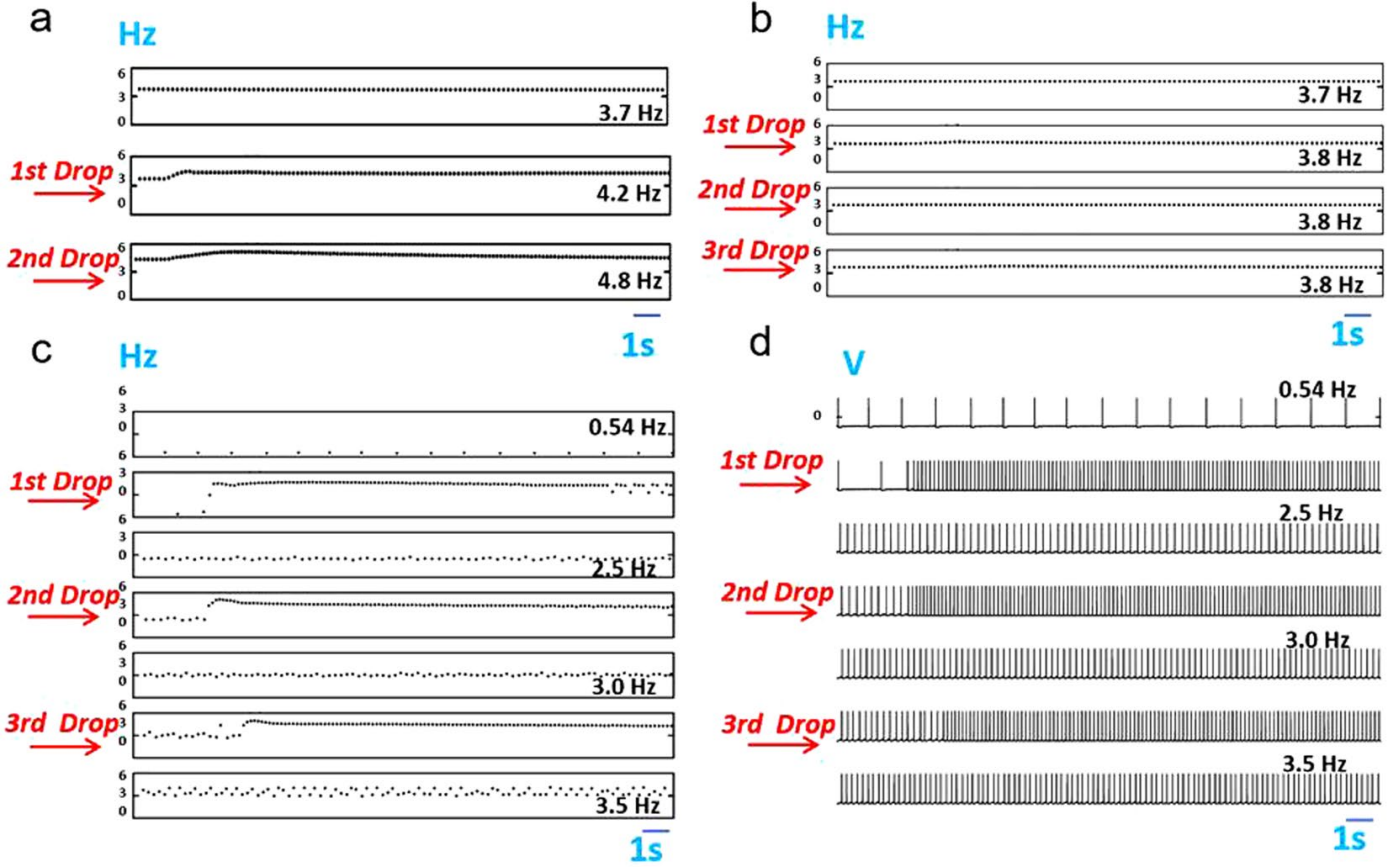
Instantaneous frequency of output pulses generated by the bioinspired glucose nanobiosensor during glucose detection. (**a**) The pulse frequency elevation was induced by sufficient amount of glucose. (0.5 mM glucose in each drop): after each drop, the total resistance of the type II cell attached to the amperometric glucose biosensor is changed and this increases the output frequency. (**b**). PBS test and analysis of selectivity of the bioinspired nanobiosensor to glucose by adding a 2-ml, 2-ml, and 4-ml PBS solution as the first, second, and third drop, respectively. (**c**) Continuous successive addition of glucose to the electrochemical cell to record the alteration of the pulse frequency (0.5 mM glucose in each drop) (**d**) Pulse frequency was modulated by detection limit amount of glucose, including the alternating frequency (0.5 mM glucose in each drop).

Figure 4(a) shows the instantaneous frequency of output pulses generated by the electrical circuit during glucose detection. The instantaneous frequency of the output pulses was the inverse of the interpulse interval between the two successive pulses. This is defined by the pulse frequency. The pulse frequency successfully increased with an increase in the glucose concentration in the electrochemical cell. In fact, the detection of glucose by GOx in the electrochemical cell increases the number of electrons shuttled by the mediator. Thus, the current increases as the glucose concentration increases. In pursuant of the current, the decrease in the resistance of the biosensor can be detected as different pulses by the type II cell; moreover, the LED starts blinking due to the alteration of the resistance as recorded by the type III cell.

The glucose detection process is a continuous process (up to 8.9 mM); therefore, the pulse frequency continuously increased as a function of glucose concentration in the electrochemical cell. In contrast, by adding phosphate buffer solution (PBS) solution to the electrochemical cell, the resistance between the working electrode and the counter electrode in the electrochemical cell remained unchanged, and the fraction between *R*_1_ and *R*_2_ was fixed in each PBS drop (Fig. 4(b)). Dropping glucose into the electrochemical cell caused oxidation at the electrode, thereby yielding a current signal (proportional to the glucose concentration) on account of the presence of the mediator. Therefore, the glucose concentration was detected as the pulse frequency. Thus, it can be assumed that the biosensor responded to the glucose and the pulse frequency was modulated by the glucose concentration.

The conditions of the bioinspired glucose biosensor were optimised by fabricating different biosensors by varying the number of drops of MWCNTs/gelatin/GOx casted into GCE to analyse the reproducibility of the obtained frequency based on thin-film thicknesses. A thinner biosensor film on GCE behaved differently. The variation of the thickness of the biosensor on GCE caused irregular alternating high and low pulse frequencies, as shown in Fig. 4(c). Figure 4(d) displays the pulse responses of the pulse generator circuit.

**Figure 5:**
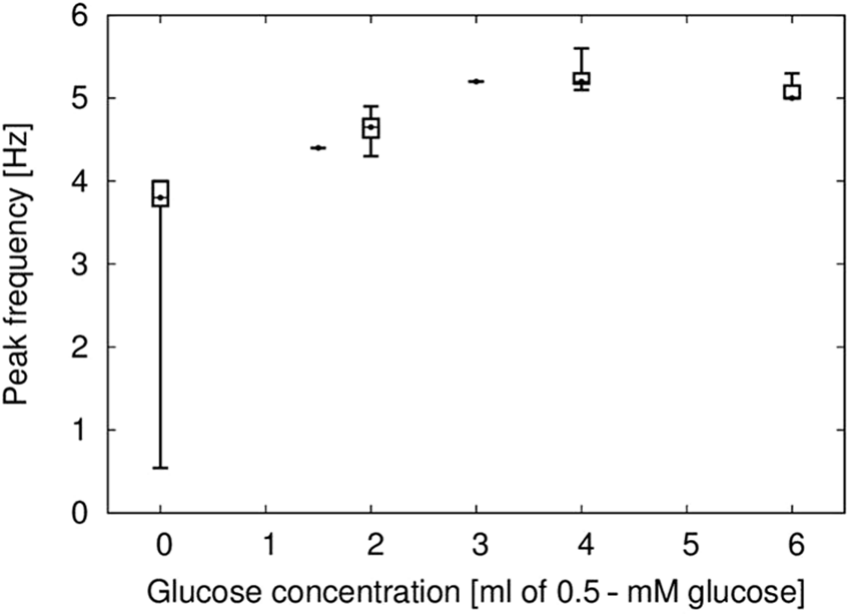
Dose-dependent response of the pulse generator as a function of glucose concentration: the strategy enabled a biosensor with the pulse generator circuit with selectivity for the detection of glucose. The frequency is gradually increases according to each drop by adding sufficient amount of glucose to the bioinspired nanobiosensor.

The reproducibility as well as the output frequency of the pulse generator as a function of glucose concentration was validated by obtaining the dose-dependent response using five different modified glassy carbon electrodes. Figure 5 displays the peak frequency due to the change in the glucose concentration in the electrochemical cell. The results indicate an increase in the frequency, which demonstrates the higher biological affinity of the pulse generator to the glucose. However, the similarity of the dose–response curves strongly suggests that the pulse generator containing the taste receptors for glucose is able to selectively recapitulate the glucose detection.

## Computational analysis of frequency alternations of the bioinspired glucose nanobiosensor induced by glucose

The resistance in the electrochemical cell was modulated by the glucose concentration and the applied voltage. Adding glucose to the electrochemical cell modulated the fraction between *R*_1_ and *R*_2_. Consequently, the applied voltage to the electrochemical cell changed. This led to the reassignment of *R*_1_. Such a tuning process automatically occurred and the output frequency approached a new stable state. If the resistance in the electrochemical cell was time variant, the output frequency might not have been derived from Eq. 4. In the present study, the output voltage, *V*_*o*_, was calculated by a numerical integration method.

Potential *V*_*t*_ is determined by the fraction between *R*_1_ and *R*_2_. Inverting input *V*_*c*_ and non-inverting input *V*_*t*_ are determined by the following equations:

5 $$\frac{d{V}_{c}}{dt}=\frac{{V}_{o}-{V}_{c}}{{R}_{t}{C}_{t}}$$

6 $${V}_{t}=\frac{{R}_{1}}{{R}_{1}+{R}_{2}}{V}_{o}$$

where *V*_*o*_ < *V*_*c*_, *R*_*t*_ = 10 kΩ; otherwise *R*_*t*_ = 100 kΩ. *R*_2_ = 2 kΩ.

By adding glucose to the electrochemical cell, *R*_1_ changed as a function of the applied voltage and the glucose concentration as follows:

7 $${R}_{1}=f([{\rm{Glu}}],{V}_{{\rm{applied}}})$$

The non-inverting input, *V*_*t*_, was directly proportional to the applied voltage (*V*_applied_). It could be assumed that the chemical reaction in the electrochemical cell has a delay in the reassignment of the resistance^28^. This can be described by the following equation:

8 $${R}_{1}(t)=\{\begin{array}{ll}{R}_{1{\rm{high}}} & \,\mathrm{if}\,{V}_{o}(t+\tau )=1.5\\ {R}_{1{\rm{low}}} & \,\mathrm{if}\,{V}_{o}(t+\tau )=-\,1.5\end{array}$$

where *τ* is a delay of the chemical reaction, and *t* is the reaction time.

A pulse-to-pulse variation of the pulse generator was evaluated by a computer simulation of the above equations. The computer program was written in the C language. The differential equation of voltage *V*_*c*_ was numerically integrated. Consequently, the output voltage of the pulse generator was obtained. The forward Euler integration method was used for the numerical integration.

The alternation of high and low pulse frequencies in the output pulse was clarified by a computer simulation of the pulse generator. The computation was performed with given values of resistance *R*_1_. Adding glucose to the electrochemical cell reduced *R*_1_ During the first 10 s, the computer simulation was performed with the initial values. In the next 10 s, the electrode resistance *R*_1_ was replaced with lower values in the pulse generator. The initial values were *R*_1 high_ = 5 kΩ and *R*_1 low_ = 50 kΩ.

**Figure 6:**
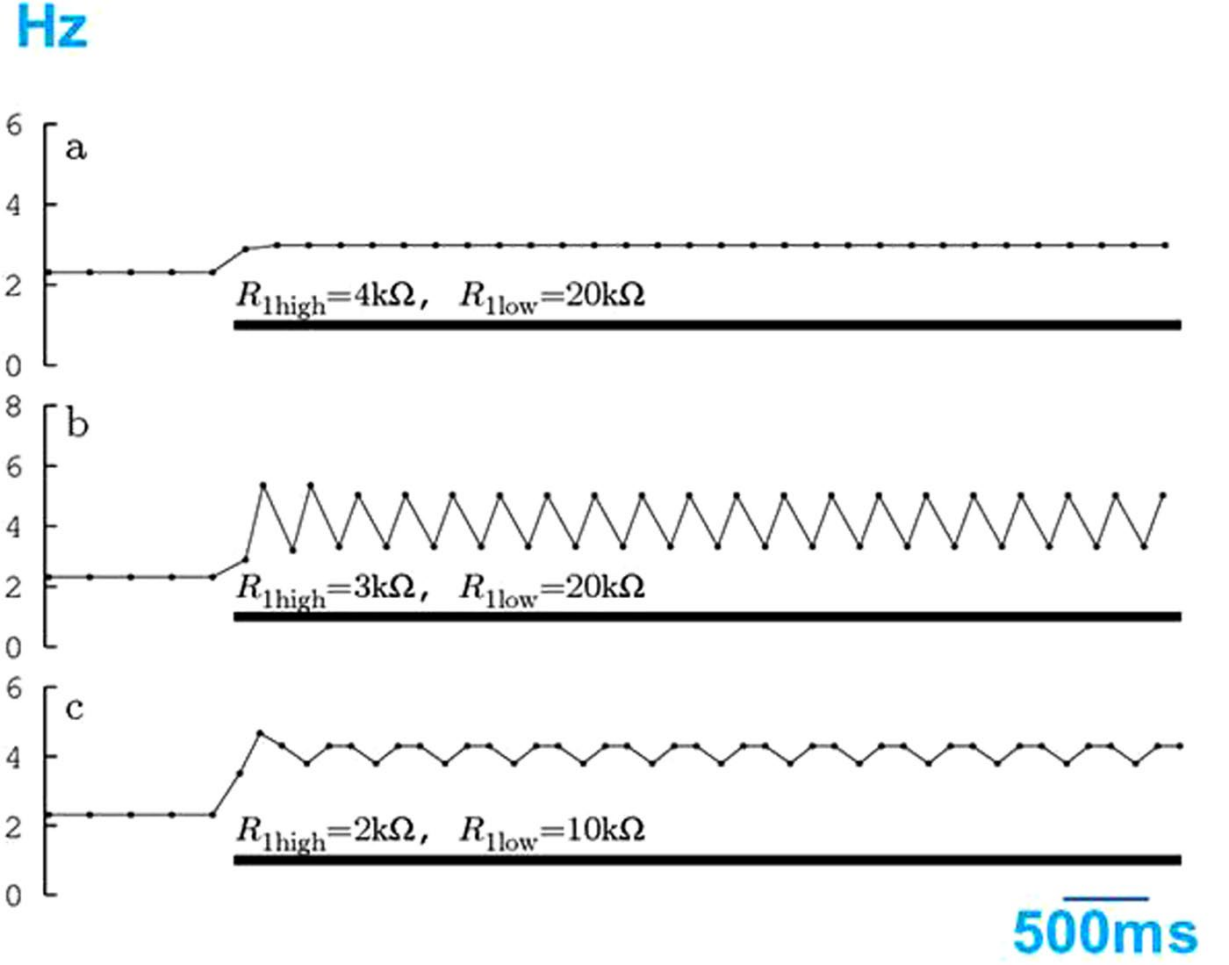
Dependence of the simulation results of the pulse generator on the electrode resistance. The thick bar below each trace represents the low resistance condition. (**a**) Reduction of the electrode resistance induces high-frequency oscillations and (**b**) Periodic alternations of high frequency pulse and low frequency pulse appear (alternative oscillations). (**c**) The output pulses display regular cycles every three pulses (period three oscillation).

Figure 6 shows the computer simulation results of the resistance changes that occurred while adding glucose to the electrochemical cell. The delay *τ* was 500 ms. A decrease in the resistance increased the pulse frequency (Fig. 6(a)). Further reductions induced periodic alternations of the pulse frequency (Fig. 6(b)) or period-three oscillation (Fig. 6(c)). The correspondence between the behaviour displayed by the mathematical model and those of the experimental observations was evident. When the resistance of the pulse generator was higher than the required resistance for the alternating pulse frequency, normal periodic pulses were achieved, whereas in the lower resistance range, the alternating pulse frequency emerged from the pulse generator instability.

The reasons for the alternation of high and low pulse frequencies may have been twofold. Firstly, decreasing the thickness of the thin film consisting of the biosensors increases the resistivity of the working electrode. Hence, each successive drop of glucose added to the electrochemical cell causes the electrons shuttled by the mediator to decrease *R*_1_ until electron transfer is complete. Subsequently, *R*_1_ rebounds to its initial value. This alternation phenomenon continues until the full detection of the glucose by the available immobilised glucose oxides on the MWCNTs/Gl composite and the finalisation of the transformation process of the electrons in the electrochemical cell. Secondly, the alternation in frequency may have been caused by the alteration of the resistance during the oxidation and reduction of the mediator. In other words, the initial resistance is defined by the axis line in the frequency plot. Each oxidation increases the frequency to above the axis line and each reduction decreases the frequency to below the axis line.

## Conclusion

A novel method to fabricate a frequency-based artificial taste receptor for glucose diagnosis using an enzymatic biosensor was proposed. The spiking property of the glucose stimulation in the neuron–like circuit was investigated. The first layer of the bioinspired glucose nanobiosensor functioned as a periodic pulse generator. The resistance of the amperometric glucose biosensor was altered on account of the glucose concentration and applied voltage. Those resistance alternations caused frequency changes in the bioinspired glucose nanobiosensor. The results clearly confirmed that the addition of glucose to the bioinspired glucose nanobiosensor changes the output oscillation frequency, causing the square-wave frequency to stabilise at a new level. Although the fabricated bioinspired glucose nanobiosensor was developed for the electrochemical detection of glucose, a potential response method can be developed for integration of the complete categorised artificial mouse tongue by the electrochemical detection of all types of detectable tastes.
